# Elevated serum alpha-1 antitrypsin is a major component of GlycA-associated risk for future morbidity and mortality

**DOI:** 10.1371/journal.pone.0223692

**Published:** 2019-10-23

**Authors:** Scott C. Ritchie, Johannes Kettunen, Marta Brozynska, Artika P. Nath, Aki S. Havulinna, Satu Männistö, Markus Perola, Veikko Salomaa, Mika Ala-Korpela, Gad Abraham, Peter Würtz, Michael Inouye

**Affiliations:** 1 Cambridge Baker Systems Genomics Initiative, Baker Heart & Diabetes Institute, Melbourne, Victoria, Australia; 2 Cambridge Baker Systems Genomics Initiative, Department of Public Health and Primary Care, University of Cambridge, Cambridge, United Kingdom; 3 Department of Clinical Pathology, The University of Melbourne, Parkville, Victoria, Australia; 4 Computational Medicine, Faculty of Medicine, University of Oulu and Biocenter Oulu, Oulu, Finland; 5 National Institute for Health and Welfare, Helsinki, Finland; 6 NMR Metabolomics Laboratory, School of Pharmacy, University of Eastern Finland, Kuopio, Finland; 7 Institute for Molecular Medicine Finland, University of Helsinki, Helsinki, Finland; 8 Population Health Science, Bristol Medical School, University of Bristol, Bristol, United Kingdom; 9 Medical Research Council Integrative Epidemiology Unit, University of Bristol, Bristol, United Kingdom; 10 Systems Epidemiology Lab, Baker Heart & Diabetes Institute, Melbourne, Victoria, Australia; 11 Department of Epidemiology and Preventive Medicine, School of Public Health and Preventive Medicine, Faculty of Medicine, Nursing and Health Sciences, The Alfred Hospital, Monash University, Melbourne, Victoria, Australia; 12 School of BioSciences, The University of Melbourne, Parkville, Victoria, Australia; 13 Research Programs Unit, Diabetes and Obesity, University of Helsinki, Helsinki, Finland; 14 Nightingale Health Ltd, Helsinki, Finland; 15 The Alan Turing Institute, London, United Kingdom; Beijing Key Laboratory of Diabetes Prevention and Research, CHINA

## Abstract

**Background:**

GlycA is a nuclear magnetic resonance (NMR) spectroscopy biomarker that predicts risk of disease from myriad causes. It is heterogeneous; arising from five circulating glycoproteins with dynamic concentrations: alpha-1 antitrypsin (AAT), alpha-1-acid glycoprotein (AGP), haptoglobin (HP), transferrin (TF), and alpha-1-antichymotrypsin (AACT). The contributions of each glycoprotein to the disease and mortality risks predicted by GlycA remain unknown.

**Methods:**

We trained imputation models for AAT, AGP, HP, and TF from NMR metabolite measurements in 626 adults from a population cohort with matched NMR and immunoassay data. Levels of AAT, AGP, and HP were estimated in 11,861 adults from two population cohorts with eight years of follow-up, then each biomarker was tested for association with all common endpoints. Whole blood gene expression data was used to identify cellular processes associated with elevated AAT.

**Results:**

Accurate imputation models were obtained for AAT, AGP, and HP but not for TF. While AGP had the strongest correlation with GlycA, our analysis revealed variation in imputed AAT levels was the most predictive of morbidity and mortality for the widest range of diseases over the eight year follow-up period, including heart failure (meta-analysis hazard ratio = 1.60 per standard deviation increase of AAT, P-value = 1×10^−10^), influenza and pneumonia (HR = 1.37, P = 6×10^−10^), and liver diseases (HR = 1.81, P = 1×10^−6^). Transcriptional analyses revealed association of elevated AAT with diverse inflammatory immune pathways.

**Conclusions:**

This study clarifies the molecular underpinnings of the GlycA biomarker’s associated disease risk, and indicates a previously unrecognised association between elevated AAT and severe disease onset and mortality.

## Introduction

The identification and characterisation of new predictive biomarkers for disease is fundamental to precision medicine [1,2]. Biomarkers discovered using systems-level technologies can be complex and heterogeneous, thus it can be challenging to pinpoint relevant biomolecular pathways. Therefore, knowledge of the underlying molecular basis for a biomarker is critical for identifying potential therapeutic targets and interventions.

Of recent interest is the GlycA biomarker, a serum NMR signal that has been shown to be highly predictive of morbidity and mortality from diverse diseases [3,4], including cardiovascular diseases [5–8], certain cancers [8–10], type II diabetes [5,11–13], liver diseases [5,14], chronic inflammatory conditions [5,8], renal failure [5], severe infections [15], and all-cause mortality [8,10]. Elevated GlycA levels are associated with inflammation arising from recent infection, injury, or chronic disease [16–21], as well as low-grade chronic inflammation that may persist for up to a decade in otherwise apparently healthy adults [15]. Interestingly, the associations between elevated GlycA and disease morbidity and mortality have been largely independent of C-reactive protein (CRP) [5,6,8–12,14,15], the standard biomarker for inflammation [22], with suggestions that GlycA better captures systemic inflammation due to its composite nature [3,13,17]. The GlycA signal is an agglomeration of at least five circulating glycoprotein concentrations: predominantly alpha-1 antitrypsin (AAT), alpha-1-acid glycoprotein (AGP), haptoglobin (HP), transferrin (TF), and alpha-1-antichymotrypsin (AACT) [16,17]. The heterogeneous composition of GlycA represents a challenge for further research towards investigating and developing molecular intervention strategies. This is compounded by the dynamic nature of each glycoprotein, each of which responds over different time scales, directions, and magnitudes as part of the inflammatory response [15–17,23]. Thus, two individuals with the same GlycA levels may have differing concentrations of each glycoprotein contributing to the NMR spectral signal. Further, high-throughput NMR spectroscopy cannot measure the concentrations of the individual glycoproteins comprising GlycA, which require the use of specialised immunoassays. However, such immunoassays are costly and time-consuming. Here, we decompose the spectral GlycA biomarker by developing imputation models for GlycA's constituent glycoproteins, then utilise these imputed molecular phenotypes to investigate associations with disease risk. Our findings provide important insights into potential intervention strategies for GlycA-associated disease and mortality risk and may lead to better disease risk stratification.

## Results

**Fig 1:**
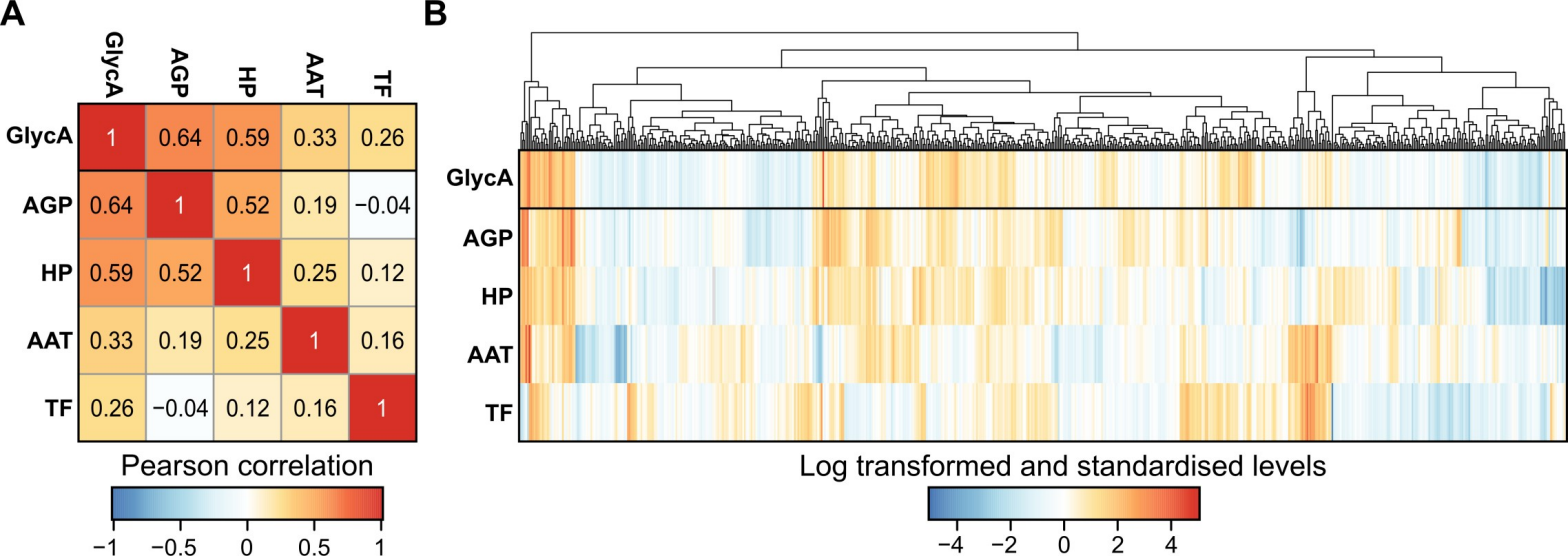
Relationship between GlycA and its constituent glycoproteins. A) Heatmap of the Pearson correlation between GlycA, AAT, AGP, HP, and TF in the 626 DILGOM07 participants with matched NMR metabolite measurements and glycoprotein assay data after log transformation and standardisation. Rows and columns have been ordered in decreasing order of correlation coefficient with GlycA. B) Heatmap of the log transformed and standardised concentrations of GlycA and each glycoprotein. Columns correspond to DILGOM07 participants, which have been hierarchically clustered (average linkage) based on their Euclidean distance calculated on their GlycA and glycoprotein measurements. Rows are ordered as in panel A.

To investigate the relationship between GlycA and its constituent glycoproteins in a population setting we utilised matched serum NMR-metabolite measures and immunoassays for AAT, AGP, HP, and TF in 626 adults previously measured in the population-based DIetary, Lifestyle, and Genetic determinants of Obesity and Metabolic syndrome 2007 study (DILGOM07) [15,24]. AACT was not available in this cohort as immunoassay measurements were performed prior to its establishment as a significant contributor to the GlycA signal by reference [17]. Consistent with our previous analysis of this data [15], all four glycoproteins were strongly positively correlated with GlycA (Fig 1A). AGP was the strongest correlate of GlycA (Pearson correlation *r* = 0.64), followed by HP (*r =* 0.59), AAT (*r* = 0.33), and TF (*r =* 0.26). There was moderate positive correlation between most glycoproteins, with a Pearson *r* range of 0.12 to 0.52, with the exception of the AGP and TF which were not correlated (*r* = −0.04) (Fig 1A). Hierarchical clustering revealed distinct clusters of individuals who had similar GlycA levels but heterogeneity in glycoprotein profiles (Fig 1B), indicating a complex relationship between GlycA and its constituent acute-phase glycoproteins and suggesting the individual glycoproteins may each differentially predict long term incident disease risk.

**Fig 2:**
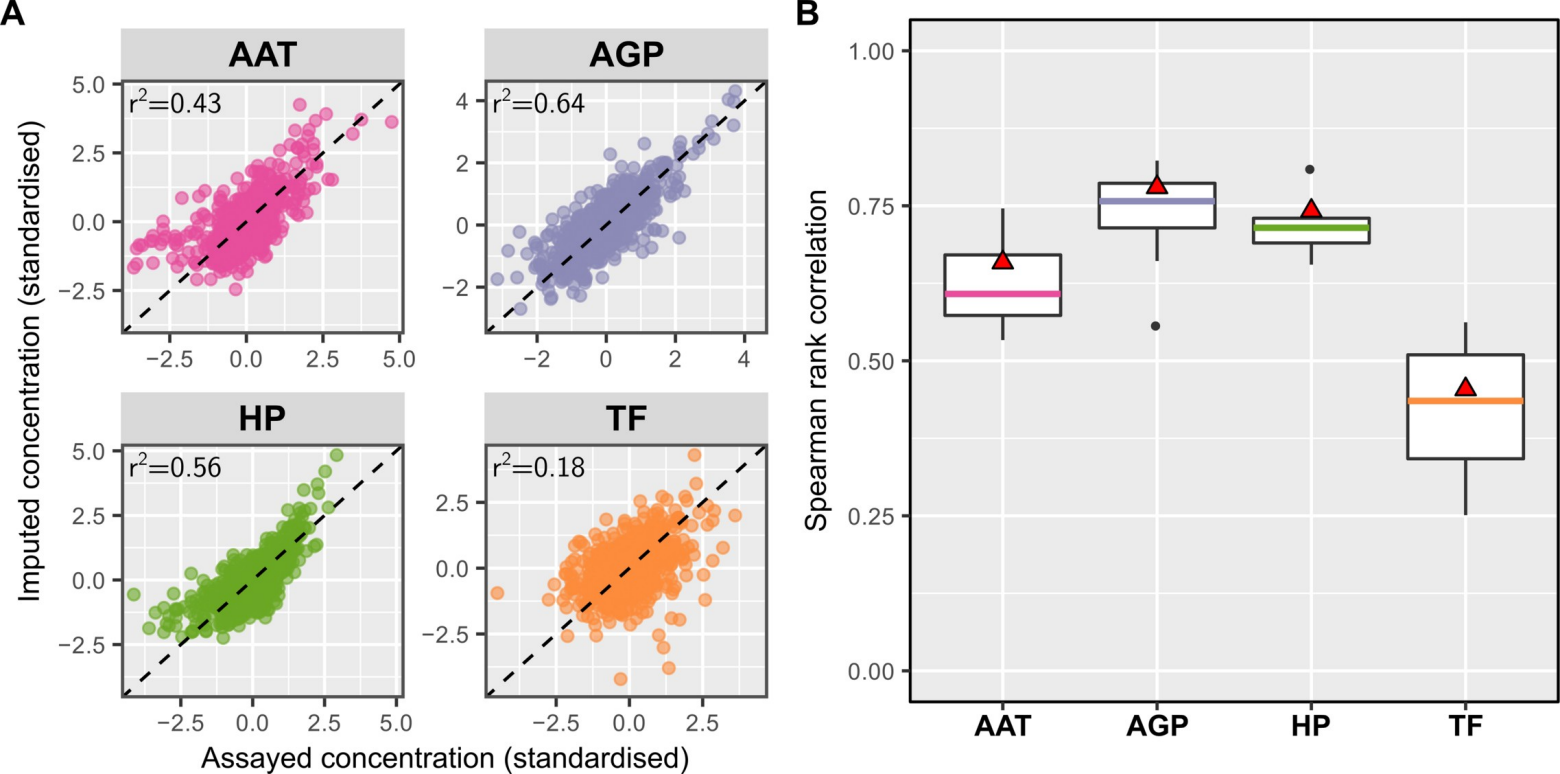
Comparison of imputation models to glycoprotein immunoassays in the 626 DILGOM07 participants with matched glycoprotein assay and metabolite quantification by NMR metabolomics. A) Comparison of the imputed glycoprotein levels (y-axes) to the immunoassayed glycoprotein levels (x-axes) after log transformation and standardisation. The r^2^ value indicates the proportion of variance in the assayed glycoprotein explained by the respective imputation models. B) Boxplots of the Spearman correlation between the imputed and observed concentrations observed in the 10-fold cross validation procedure used for model training. Red triangles show the Spearman correlation between the predicted and observed concentrations in panel A (detailed in S2 Table).

We utilised machine learning together with the matched serum NMR-metabolite measures and immunoassays for AAT, AGP, HP, and TF in 626 DILGOM07 participants to develop imputation models for the concentrations of each glycoprotein. Lasso regression [25,26] was used to find the optimal subset of features and corresponding weights that most accurately predicted each glycoprotein. A 10-fold cross-validation procedure was used to train each lasso regression model to reduce overfitting and estimate model accuracy (S1 Fig, Methods). In total, 149 metabolic measurements quantified via NMR (S1 Table) along with participant age, sex, and body mass index (BMI) were included as features to the model training procedure. The imputation models for AAT, AGP, HP, and TF explained 43%, 64%, 56% and 18% of their variation (r^2^), respectively, (Fig 2A) and comprised 18, 23, 27, and 9 input features, respectively (S1 Models).

Comparison of each imputation model’s predicted levels to the observed immunoassayed levels in DILGOM07 (Fig 2A) along with cross-validation estimates of the Spearman correlation (ρ) obtained during model training (Fig 2B and S2 Table and S1 Methods) indicated the imputation models for AAT (Spearman’s ρ = 0.63), AGP (Spearman’s ρ = 0.74), and HP (Spearman’s ρ = 0.71) were sufficiently accurate for downstream analysis. In contrast, the imputation model for TF was substantially less accurate (Spearman’s ρ = 0.42 and variation r^2^ = 0.18; Fig 2 and S2 Table) so was not taken forward for electronic health record association analyses.

**Fig 3:**
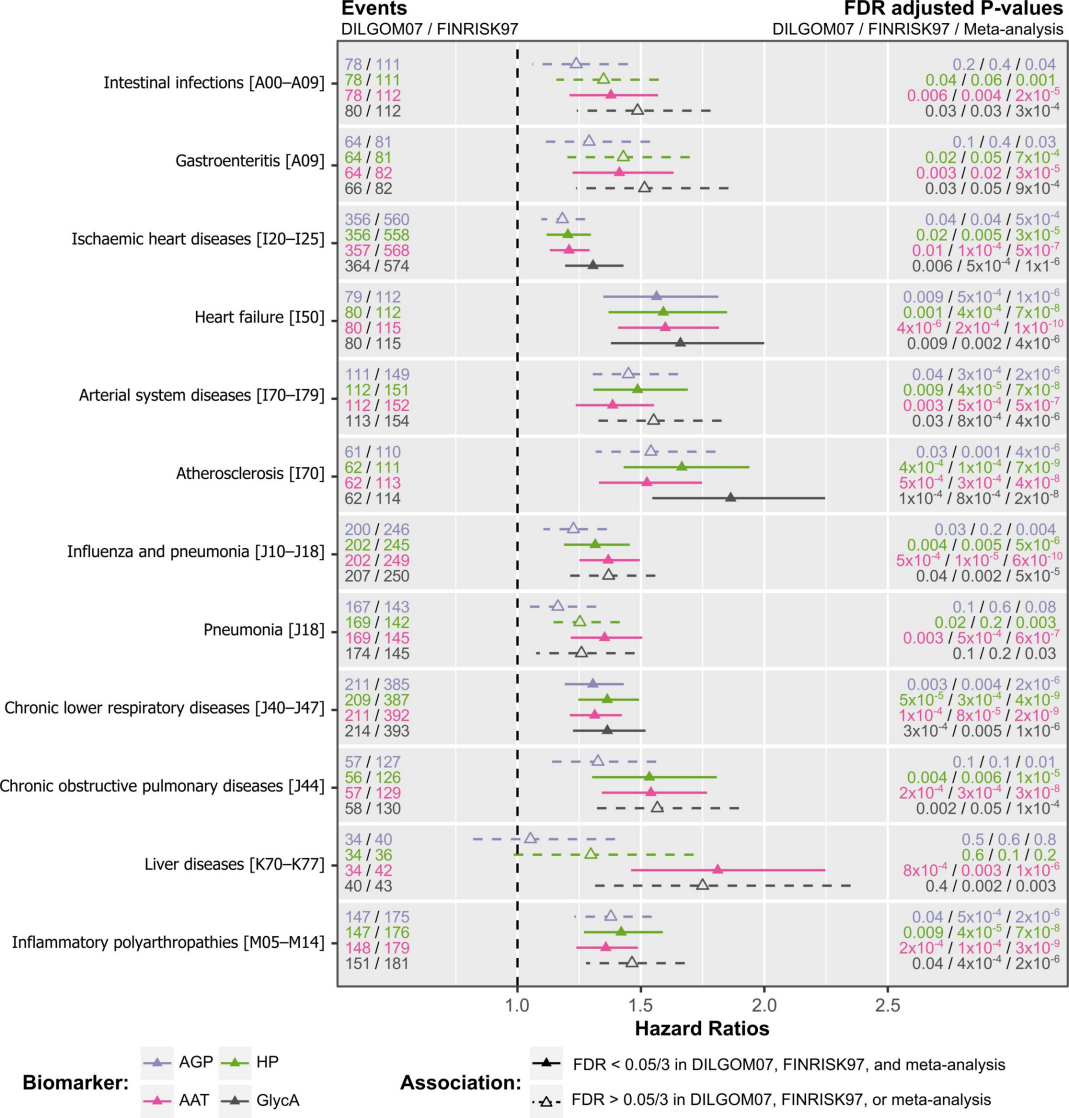
Glycoprotein associated risks of disease and mortality. Comparison of Cox proportional hazard ratios (triangles) for the first diagnosis occurrence (hospitalisation or mortality) conferred per standard deviation increase of AAT, HP, AGP, or GlycA in inverse-variance weighted fixed effects meta-analysis of DILGOM07 and FINRISK97. Bars around each hazard ratio indicate the 95% confidence interval. Diagnosis data were analysed for a total of 351 outcomes with >20 events in both DILGOM07 and FINRISK97 over a matched 8-year follow-up period. Models were fit using age as the time scale and adjusting for sex, smoking status, BMI, blood pressure, alcohol consumption, prevalent disease prior to baseline (Methods), and previously identified biomarkers for 5-year risk of all-cause mortality (citrate, albumin, and VLDL particle size). Only outcomes with a significant and replicable association with at least one of AAT, HP, or AGP are shown (Storey-Tibshirani FDR adjusted P-value < 0.05/3, adjusting for the three glycoproteins, in DILGOM07, FINRISK97, and meta-analysis). Associations which were significant and replicable are shown with solid hazard ratios and 95% confidence intervals. The alphanumeric codes in the square brackets indicate the ICD10 code or ICD10 disease group for each diagnosis. The number of events in DILGOM07 and FINRISK97 are shown to the left of each hazard ratio for each outcome. Different numbers of events for the same outcome between biomarkers arise from differences in the number of samples for which each glycoprotein was successfully imputed (Methods). Hazard ratios fit separately in DILGOM07 and FINRISK97 along with comparison to the hazard ratios calculated from the immunoassayed AAT, HP, and AGP measurements can be found in S2 Fig. Hazard ratios for all tested outcomes are detailed in S3 Table.

We next imputed AAT, AGP and HP concentrations in 4,540 DILGOM07 participants and 7,321 participants from the population-based FINRISK study 1997 (FINRISK97) [27–29], then analysed linked electronic hospital records over a matched 8-year follow-up period (Methods). Baseline cohort characteristics are described in Table 1. We observed strong, consistent, and replicable associations (False Discovery Rate adjusted P-value < 0.017, additional Bonferroni correction for the three glycoproteins) between each of AAT, AGP, and HP and increased risk of morbidity and mortality for a diverse range of disease outcomes (Figs 3 and S2), consistent with associations seen for GlycA itself [5]. Importantly, hazard ratios calculated from the imputed measurements were consistent with those from directly assayed glycoproteins in the 630 DILGOM07 participants in which they were measured (S2 Fig), indicating that the imputation models remained similarly accurate in the full DILGOM07 and FINRISK97 cohorts. In meta-analysis of DILGOM07 and FINRISK97, hazard ratios (HRs) were only slightly attenuated when adjusting for CRP (S3 Fig).

**Table 1:** Cohort characteristics.

	DILGOM07 (Model training dataset)	DILGOM07 (Full dataset)	FINRISK97
**Collection year**	2007	2007	1997
**Number of participants**	626	4,540	7,321
**Number (and %) of women**	328 (53%)	2,387 (53%)	3,644 (50%)
**Mean age in years (and range)**	53 (25–74)	52 (25–74)	48 (25–74)
**Follow-up time**	8 years	8 years	8 years
**Body mass index (kg/m**^**2**^**)**	26.80 ± 4.66	27.2 ± 4.8	26.6 ± 4.5
**GlycA (mmol/L)**	1.30 ± 0.18	1.30 ± 0.20	1.41 ± 0.25
**Glycoprotein assays (# participants)**			
** AAT (mg/L)**	1.19 ± 0.20 (N = 615)	1.19 ± 0.20 (N = 626)	-
** AGP (mg/L)**	789 ± 203 (N = 615)	793 ± 205 (N = 626)	-
** HP (mg/L)**	1.09 ± 0.49 (N = 614)	1.10 ± 0.50 (N = 622)	-
** TF (mg/L)**	2.65 ± 0.38 (N = 615)	2.66 ± 0.38 (N = 626)	-
**Imputed glycoproteins (# participants)**			
** AAT (mg/L)**	1.18 ± 0.11 (N = 615)	1.16 ± 0.09 (N = 4,496)	1.29 ± 0.11 (N = 7,246)
** AGP (mg/L)**	779 ± 145 (N = 615)	786 ± 142 (N = 4,474)	832 ± 178 (N = 7,151)
** HP (mg/L)**	1.04 ± 0.40 (N = 614)	1.00 ± 0.33 (N = 4,491)	1.14 ± 0.46 (N = 7,194)
** TF (mg/L)**	2.63 ± 0.10 (N = 615)	-	-

Data are reported as the mean ± standard deviation (s.d.) unless otherwise indicated.

Consistent with previous studies of GlycA [15–17], AGP was the most strongly correlated glycoprotein with GlycA (Spearman ρ = 0.65; S4 Table). Despite AGP levels explaining the most variance in GlycA levels, which would suggest it should consequently be the strongest biomarker for incident disease, we found that imputed AAT was significantly associated with risk of hospitalisation or death for substantially more outcomes (Fig 3). Elevated concentrations of imputed AAT were associated with increased 8-year risk from a wide range of disease classifications, including liver diseases (Hazard Ratio = 1.81 per standard deviation increase in AAT, 95% Confidence Interval = 1.46–2.25, False Discovery Rate adjusted P-value = 1×10^−6^), heart failure (HR = 1.60, 95% CI = 1.41–1.82, FDR = 1×10^-10^), and chronic obstructive pulmonary disease (HR = 1.54, 95% CI = 1.34–1.77, FDR = 3×10^−8^) (full list given in Fig 3). In contrast, imputed AGP was significantly associated with increased risk from only two outcomes: heart failure (HR = 1.56, 95% CI = 1.35–1.81, FDR = 1×10^−6^) and chronic lower respiratory diseases (HR = 1.31, 95% CI = 1.19–1.43, FDR = 2×10^−6^) (Fig 3). Together with the complex relationships between the glycoprotein levels and GlycA (Fig 1B), this indicates that variation in AAT levels were more predictive of future disease than variation in AGP levels.

**Fig 4:**
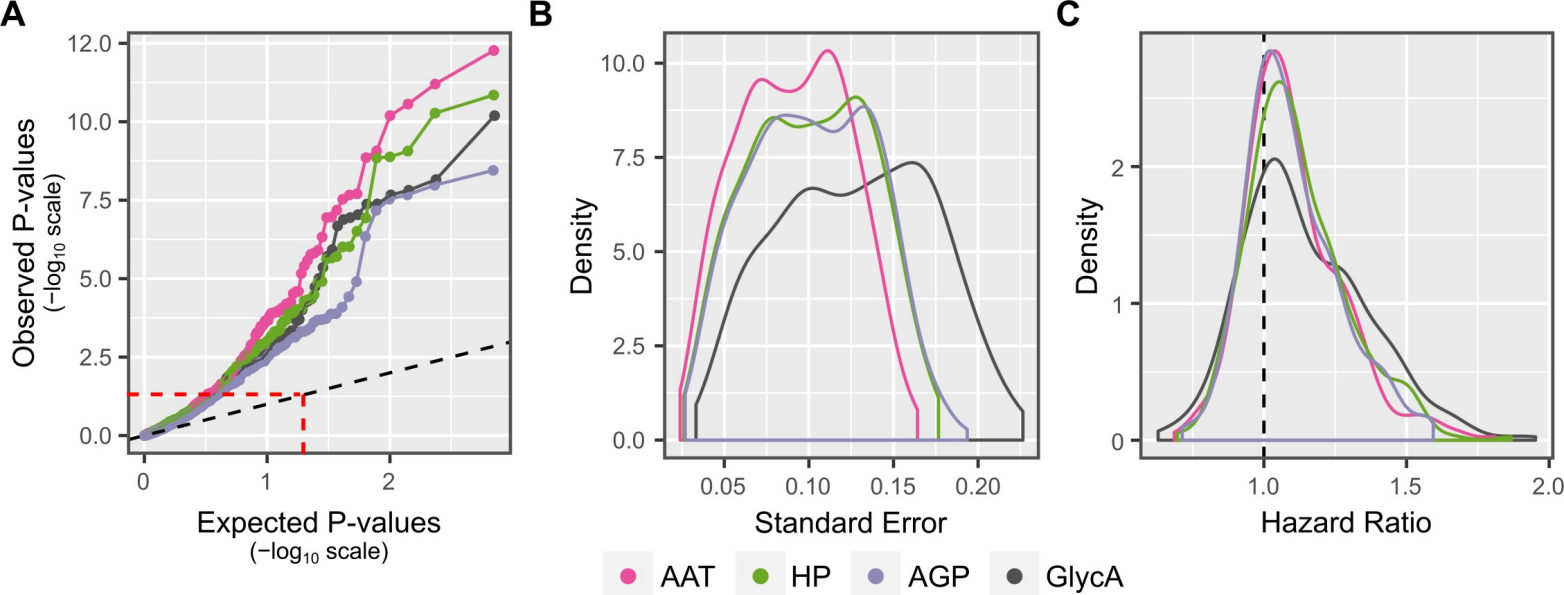
Comparison of biomarkers across all outcomes in meta-analysis of DILGOM07 and FINRISK97. A) Quantile-Quantile plots of distributions of hazard ratio estimate P-values (y-axis) compared to distribution of expected P-values under the null hypothesis that the corresponding biomarker is not associated with any outcome (x-axis). Hazard ratio estimate P-values are shown after adjustment for multiple testing using the Storey-Tibshirani FDR method. The dashed line indicates the location where p-values would fall if the observed distribution was identical to the null distribution. Points above the red dashed line indicated hazard ratios with FDR adjusted P < 0.05 in the meta-analysis, while points above the blue dashed line indicate hazard ratios with FDR adjusted P < 0.05/3 in the meta-analysis. B) Density plots comparing each biomarker’s distribution of hazard ratio standard errors across all outcomes. C) Density plots comparing each biomarker’s distribution of hazard ratios across all outcomes.

Sensitivity analysis showed that the wide range of associations between imputed AAT and outcomes was robust to the significance threshold (Fig 4A). Furthermore, AAT hazard ratios tended to have the smallest standard errors across all tested outcomes (Fig 4B). AGP was associated with the fewest outcomes regardless of significance threshold (Fig 4). Among the significant and replicable associations, AAT was the strongest predictor for all but four outcomes, for which HP was the strongest predictor (Fig 3). HP was the strongest predictor of chronic lower respiratory diseases (HR = 1.36, 95% CI = 1.25–1.49, FDR = 4×10^−9^), inflammatory polyarthropathies (HR = 1.42, 95% CI = 1.27–1.59, FDR = 7×10^−8^), and atherosclerosis (HR = 1.67, 95% CI = 1.43–1.94, FDR = 7×10^-9^) as well as the broader grouping of all arterial system diseases (HR = 1.49, 95% CI = 1.31–1.69, FDR = 1×10^-9^).

While our focus here is on identifying the molecular glycoprotein associations with disease, we also performed a comparison with the GlycA NMR signal. Compared to the GlycA biomarker itself, imputed AAT was more strongly associated with a wider range of outcomes regardless of choice of significance threshold (Fig 4A). However, the GlycA HRs tended to be stronger than those for both AAT and HP, but with larger standard errors (Figs 4 and 3). This suggests that each glycoprotein predicts different but overlapping components of disease risk, consistent with the overlapping elevated levels of each glycoprotein observed in Fig 1B, with GlycA levels capturing this risk in aggregate.

With the preponderance of AAT-associated incident disease risk and previously observed associations between GlycA and systemic inflammation [15], we investigated whether, and to what extent, elevated AAT was associated with inflammatory processes. We used whole blood transcriptome data with matched serum AAT immunoassay data in 518 DILGOM07 participants to identify transcriptional signals in circulating immune cells associated with elevated AAT. We utilised Gene Set Enrichment Analysis (GSEA) [30,31] to identify pathways enriched for AAT-associated differential expression, and additionally tested for association with AAT the coordinated summary expression profiles of previously identified transcriptional network modules (Methods). Two sets of network modules were tested: (1) 20 modules of functionally coexpressed genes we previously identified in DILGOM07 [15,32–34] and replicated in an independent cohort [34], and (2) 346 blood transcript modules identified by Li *et al*. from 30,000 blood samples across 500 studies [35].

GSEA analysis revealed a wide variety of immune response pathways were significantly enriched for genes upregulated with elevated AAT (FDR<0.05; Tables 2 and S5). Elevated serum AAT protein levels were associated with increased transcription of genes involved in reactive oxygen species (FDR adjusted P = 2×10^−3^), immune response initiation pathways (*e*.*g*. IL6/JAK/STAT signalling, FDR adjusted P = 0.02), innate immune response pathways (*e*.*g*. genes localising to phagocytic vesicles, FDR adjusted P = 8×10^−3^), adaptive immune response pathways (*e*.*g*. Toll-like receptor signalling pathway, FDR adjusted P = 0.04), and numerous cytokine regulation pathways (Tables 2 and S5).

**Table 2:** Highlighted gene sets significantly enriched for genes associated with AAT.

Collection	Gene set	Size	NES	FDR
Hallmark	Reactive oxygen species pathway	43	2.21	0.002
Hallmark	TNFa signaling via NFkB	194	1.92	0.01
Hallmark	PI3K/AKT/mTOR signaling	101	1.88	0.02
Hallmark	IL6/JAK/STAT3 signaling	81	1.94	0.02
Hallmark	Apoptosis	154	1.85	0.02
KEGG	Toll-like receptor signaling pathway	96	2.05	0.04
Reactome	Toll receptor cascades	102	1.98	0.04
GO:BP	Cytokine production involved in immune response	17	2.24	0.006
GO:BP	T cell differentiation involved in immune response	28	1.96	0.03
GO:BP	Antimicrobial humoral response	43	1.97	0.03
GO:BP	Defense response to fungus	35	1.93	0.04
GO:BP	Regulation of innate immune response	325	1.86	0.05
GO:BP	Phagocytosis engulfment	17	1.87	0.05
GO:BP	Antigen processing and presentation of peptide antigen via MHC class I	86	1.86	0.05
GO:BP	Negative regulation of viral process	84	1.85	0.05
GO:BP	Negative regulation of immune response	113	1.85	0.05

A selection of the gene sets that were significantly enriched for AAT-associated differential expression (Methods). See S5 Table for a full listing of all 139 gene sets significantly enriched for AAT associated genes. Gene sets shown here were selected to highlight the association between elevated AAT and increase expression of diverse immune response pathways. A gene set was considered significantly enriched for AAT associated genes if its Benjamini-Hochberg FDR adjusted permutation test P-value for enrichment was < 0.05 (FDR correction performed within each gene set collection separately). The tested gene set collections included Hallmark pathways, KEGG pathways, Reactome pathways, GO biological process (GO:BP) terms, GO molecular function (GO:MF) terms, and GO cellular compartments (GO:CC). Size: number of genes on the Illumina HT-12 array annotated for the corresponding gene set. NES: enrichment score normalized by gene set size in a permutation procedure (Methods).

Of our replicable DILGOM07 whole blood coexpression modules, three were significantly associated AAT (P<0.0025; Bonferroni adjusting for the 20 tested modules) (S6 Table). Two modules previously characterised as general immune function modules [34] had increased expression with elevated AAT, and one module characterised here (S7 Table) for RNA processing function had decreased expression with elevated AAT. Elevated AAT was nominally associated (P < 0.05) with increased expression the neutrophil antimicrobial peptide module [15], the viral response module [34], and the general cell signalling response (S7 Table), along with decreased expression of the B cell activity module [34] and the cytotoxic cell-like module [34].

Of the 346 blood transcript modules [35], 30 were Bonferroni significant (P < 1.45×10^−4^) and 115 were nominally significant (P < 0.05) (S8 Table). All 30 of the Bonferroni significant modules had elevated expression with elevated AAT. These modules were enriched for activity of a wide range of immune cells, both innate and adaptive, including neutrophils, myeloid cells, monocytes, dendritic cells, T-cells, and B-cells, along with cell signalling pathways involved in immune response. The module most strongly associated with serum AAT was “immune activation–generic cluster” with a 0.23 standard deviation increase in expression per standard deviation increase in AAT (P = 1×10^−7^).

Overall the three transcriptional analyses were consistent, pointing toward the increased expression of overall immune response rather than a specific type of immune response or inflammatory pathway with elevated serum AAT.

## Discussion

GlycA is an NMR-based biomarker predictive of morbidity and mortality from diverse disease outcomes [3–15]. It is composed of the concentrations of multiple circulating glycoproteins [15–17], each of which respond to myriad inflammatory stimuli [23]. Using circulating NMR-metabolite measures, we have developed accurate imputation models for concentrations of AAT, HP, and AGP; three of the major contributors to the GlycA signal. To investigate the molecular underpinnings of the GlycA biomarker, we imputed AAT, HP, and AGP concentrations in 11,861 generally healthy individuals from two population-based cohorts and analysed linked electronic health records over an 8-year follow-up period. Of GlycA’s constituent glycoproteins we found that AAT, rather than AGP, best explained overall future disease risk.

AAT represents a promising molecular focus for follow-up studies due to its long and established history in research, well-characterised genetic variants with large effects, widely utilised diagnostic assay, and approved therapeutics. Genetic variants in AAT, such as the Z-allele, are well-known to cause AAT deficiency, which is characterised by unusually low levels of serum AAT that cause increased risk of chronic obstructive pulmonary disease/emphysema, and liver cirrhosis [36–39]. Increased risk of chronic obstructive pulmonary disease/emphysema in AAT deficient patients is caused by insufficient inhibition of neutrophil elastase in neutrophils in the lungs [37]. Increased risk of liver cirrhosis is caused by accumulation of AAT in the liver, where the majority of AAT is produced, due to reduced migration of AAT to circulation from the liver [37]. Studies have also found AAT deficiency in individuals with rheumatoid arthritis and type II diabetes [40,41], suggesting AAT deficiency may also predispose individuals to a range of inflammation-linked disorders. Interestingly, here we found that *increased* AAT levels were predictive of morbidity and mortality for myriad common chronic diseases, suggesting that there exists a healthy window of serum AAT concentration which denotes minimal future disease risk.

Although genetically-reduced AAT levels have been shown to be causal for disease risk, the aetiological mechanisms (insufficient inhibition of neutrophil elastase and reduced migration of AAT from the liver to circulation) are unlikely to be present in individuals with increased imputed AAT. AAT is an acute-phase reactant with concentrations rising 3–4x above basal levels with inflammation due to tissue injury, infection or other exogenous insult, and may not return to normal levels for up to 6 days [23,36,42]. AAT has been found to have immunomodulating effects, and its role in regulating inflammation is being increasingly understood [43]. GlycA itself also exhibits acute-phase characteristics, although fold-increases in concentrations are rarely observed, and we have previously found that increased GlycA levels in population-based cohorts are associated with a low-grade inflammatory state in otherwise apparently healthy adults that likely persists for up to a decade [15]. Our transcriptional analysis showed a systemic increase in gene expression for inflammatory immune processes correlated with elevated AAT. Since the cohort analysed here was population-based, this systemic increase in immune system activity is unlikely to reflect acute inflammation but rather is consistent with the presence of low-grade inflammation in individuals with elevated AAT.

Chronic inflammation itself contributes to the pathophysiology of common chronic diseases and development of anti-inflammatory therapies have been of interest for reducing inflammation in order to slow disease progression [44–48]. For example, recent clinical trials found an anti-inflammatory Canakinumab, a monoclonal antibody for IL-1β, significantly reduced incidence of recurrent cardiovascular events as well as lung cancer in patients with previous myocardial infarction and elevated CRP [47,48]. Therapeutic administration of AAT (e.g. prolastin) is being trialled to reduce chronic inflammation for preventing the development and progression of type I diabetes, rheumatoid arthritis, and allograft rejection [49]. While we cannot make inferences about causality, our findings suggest that, if these trials are successful, an AAT therapy may have wide applicability across a range of diseases, including cardiovascular diseases. On the other hand, our results also suggest that AAT therapy may lead to increased adverse infection events as observed in the Canakinumab trial [47,48] and dosages would need to be carefully tuned.

Our study has several limitations. Although our results suggest that alpha-1 antitrypsin is a major predictive component of the GlycA biomarker, these results are based on imputed molecular measures, and thus regression dilution (bias towards the null as measurement noise increases) may be affecting our results. However, since the imputation model for AAT had greater noise than those for AGP and HP and we observed no difference in overfitting between the three models, we do not expect that regression dilution is substantially affecting our conclusions. In addition, we cannot preclude significant associations between elevated TF or AACT and morbidity and mortality risk, for which we were unable to develop accurate imputation models. We were unable to determine whether elevated AAT was causal of either the associated disease outcomes or the upregulation of inflammatory processes in the transcriptional analyses. We sought to use Mendelian Randomisation to help clarify whether any causal relationship exists, however, we were unable to find any suitable variants to use as instruments. We could find only two studies reporting protein QTLs for elevated serum AAT levels. A GWAS study of two Japanese populations totalling 9,359 people reported three *trans*-pQTLs for AAT; all missense variants in genes upstream of AAT [50]. A proteomics study of 1,000 Germans from the KORA cohort, identified a single *cis-*pQTL for increased serum AAT, but this did not replicate in the study replication cohorts [51]. Due to lack of replication of all four variants we concluded there was insufficient evidence for their use as instruments in a Mendelian Randomisation analysis. We could not find any reports of *cis*-eQTLs for upregulation of *SERPINA1* expression in the liver (the source of the majority of serum AAT). GWAS with larger sample sizes of serum AAT levels or liver gene expression will be needed to properly investigate causality through Mendelian Randomisation analysis.

The results of our study suggest several fruitful avenues for follow-up. The widespread availability of robust and cost effective clinical assays measuring serum AAT concentrations for diagnosis of AAT deficiency offer a potential avenue for biomarker translation. For this further studies for each individual disease would be necessary to investigate the risk prediction of the clinical assays for serum AAT and determine appropriate thresholds for clinical decision making in the context of any existing clinical risk prediction scores. The question of whether elevated serum AAT plays a causal role in future morbidity or mortality also remains to be resolved. One avenue to do so is through GWAS of assayed serum AAT levels or liver gene expression, which would enable Mendelian Randomisation analyses if genetic variants leading to elevated AAT levels are discovered. Experimental studies could also investigate the role and potential molecular mechanisms of elevated serum AAT in chronic inflammation and disease aetiopathogenesis.

This study demonstrates the power of machine learning for imputation of biomolecules for electronic health record-driven association analysis. Our results uncover a previously unrecognised relationship between elevated AAT, increased inflammation, and the risk of morbidity and mortality across a wide spectrum of common chronic diseases.

## Methods

### Study cohorts

In this study, we analysed data from two population-based cohorts. All cohort participants provided written informed consent. Protocols were designed and performed according to the principles of the Helsinki Declaration. Data protection, anonymity, and confidentiality have been assured. Ethics for the DILGOM07 and FINRISK97 cohort studies were approved by the Coordinating Ethical Committee of the Helsinki and Uusimaa Hospital District.

The 2007 collection of the Dietary, Lifestyle, and Genetic determinants of Obesity and Metabolic syndrome study (DILGOM07) cohort is an extension of the 2007 collection of FINRISK: a cross-sectional survey of the working age population in Finland conducted every 5 years [27,28]. In DILGOM07, a detailed follow-up of 5,024 individuals was conducted to collect blood samples for omic profiling, physiological measurements, and detailed surveys of lifestyle, psycho-social, and clinical questions to study the factors leading to obesity and metabolic syndrome [24]. Serum NMR profiling was conducted for 4,816 participants; AAT, AGP, HP, and TF were measured by immunoassays for 630 participants [15]; and whole blood microarray profiling was available for 518 participants [32,33]. A total of 626 participants had matched glycoprotein assay and NMR data, and 518 participants had matched glycoprotein assay and gene expression data.

The 1997 collection of the National FINRISK study (FINRISK97) cohort contains 8,446 individuals who responded of 11,500 randomly recruited from the five major regional and metropolitan areas in Finland to monitor the health of the adult population (aged 25–74) [27,28]. Serum NMR profiling was conducted for 7,602 participants with adequate serum sample available [29]. Importantly, each FINRISK collection is an independent survey; the DILGOM07 and FINRISK97 cohorts are independent of one another.

### Data quantification, processing, and quality control

Venous blood samples were collected from participants in both cohorts. For DILGOM07 venous blood was drawn after an overnight fast. For FINRISK97 the median fasting time was five hours. Serum samples were subsequently aliquoted and stored at –70C.

Concentrations of circulating AAT, AGP, HP, and TF were quantified from serum samples from 630 DILGOM07 participants (626 for HP) as previously described [15] using module analysers and Roche Tina-quant turbidimetric immunoassays. The intra-individual coefficient of variation was <3% for all four assays.

Concentrations of 228 circulating metabolites, proteins, amino acids, lipids, lipoproteins, lipoprotein subclasses and constituents, and relevant ratios were quantified by NMR metabolomics from serum samples for 4,816 DILGOM07 participants and 7,602 FINRISK97 participants. Experimental protocols including sample preparation and spectroscopy are described in reference [52]. NMR experimentation and metabolite quantification of serum samples were processed by the 2016 version of the Nightingale platform (Nightingale Health Ltd; https://nightingalehealth.com/) using a Bruker AVANCE III 500 MHz ^1^H-NMR spectrometer and proprietary biomarker quantification libraries. NMR measurements with irregular concentrations were removed and concentrations below lower detection limits set to zero by the Nightingale quality control pipeline. To facilitate log transformation, we set all zero NMR measurements to the minimum value of their respective molecular species in each cohort to approximate their lower detection limits.

Concentrations of high-sensitivity C-reactive protein (CRP) were quantified from serum samples for 4,816 DILGOM07 participants and 7,599 FINRISK97 participants using a latex turbidimetric immunoassay kit with an automated analyser.

Genome-wide gene expression profiling of whole blood for 518 DILGOM07 participants was performed as previously described [15,32,33]. Briefly, stabilised total RNA was obtained using the PAXgene Blood RNA system using the manufacturer recommended protocol. RNA integrity and quantity was evaluated for each sample using an Agilent 2100 Bioanalyser. RNA was then hybridised to Illumina HT-12 version 3 BeadChip arrays. Biotinylated cRNA preparation and BeadChip hybridisation were performed in duplicate for each sample. Microarrays were background corrected using the Illumina BeadStudio software. Probes mapping to erythrocyte globin components, non-autosomal chromosomes, or which hybridised to multiple genomic positions >10Kb apart were excluded from the analysis. Probe expression levels were obtained by taking a weighted bead-count average of their technical replicates then taking a log_2_ transform. Finally, expression levels for each sample were quantile normalised.

### Glycoprotein composition of GlycA in a population setting

Concentrations of AAT, AGP, HP, TF, and GlycA were natural log transformed and standardized in the 626 DILGOM participants with matched glycoprotein assay and NMR-metabolite data, then the Pearson correlation coefficients were calculated between all five measurements (Fig 1A). The 626 DILGOM participants were hierarchically clustered using the complete linkage method based on the Euclidean distance measured on their natural log transformed and standardized AAT, AGP, HP, TF, and GlycA levels (Fig 1B) using the hclust function and the pheatmap package version 1.0.10 in R version 3.4.2.

### Imputation model training

Lasso regression models were fit in the DILGOM07 participants to determine the contributions of the NMR-based biomarkers, participant age, sex, and BMI that best predicted the concentrations of each glycoprotein. Samples with any missing NMR data (N = 11, 1.8%) were excluded. Consequently, all derived ratios in the NMR data were excluded from the analysis due to increased missingness arising from low concentration measurements in their numerator or denominator. In total, 149 NMR measurements (S1 Table) were included in each lasso regression. In total, 615 individuals had matched glycoprotein and completed NMR metabolite data (N = 611 for HP). Age was standardised, and each glycoprotein, NMR-metabolite measure, and BMI were log transformed and standardised when fitting the lasso regression models. The models were fit using the glmnet package [53] version 2.0–2 in R version 3.1.3.

To reduce overfitting of the models to the 615 DILGOM07 participants (hereby “training cohort”), a 10-fold cross-validation procedure was used to tune the lasso regression λ penalty, which determined how many variables were included in the final imputation models for each glycoprotein (S1 Fig). In this procedure, the training cohort was randomly split into 10 groups, and a sequence of 100 λ values was generated by the cv.glmnet function in the glmnet R package. For each of these 100 λs a lasso regression was fit to each possible 9/10^ths^ of the data and the resulting model used to predict the glycoprotein concentration in the remaining 1/10^th^ of the data. To compare the accuracy of the model fit by each λ, the mean-square error (MSE) was calculated as the mean of squared difference between the predicted and observed glycoprotein in each test-fold (S1 Fig). To obtain the final imputation models (S1 Models; R package at https://github.com/sritchie73/imputegp) a lasso regression model was fit to the NMR-metabolite measures, age, sex, and BMI, for the full training cohort using the largest λ penalty with an average MSE within one standard error of the smallest average MSE in the cross-validation procedure. This λ was selected as it produced the simplest possible model for each glycoprotein with a comparable average MSE to the smallest average MSE given the uncertainty in the average MSE estimate, thus further reducing model overfitting [53].

To evaluate imputation model accuracy, the Spearman’s rank correlation coefficient (hereby Spearman correlation) was used to quantify the similarity of the imputed and immunoassayed levels of each glycoprotein (Fig 2B and S2 Table). The Spearman correlation provides an indicator of how well the imputation models are likely to distinguish between many individuals with different glycoprotein concentrations after the standard statistical treatment of normalisation and standardisation when imputing each glycoprotein in another dataset. Estimates of the Spearman correlation given in the text were obtained by taking their averages across the 10-fold cross-validation procedure in which the Spearman correlation were calculated by comparing the imputed and immunoassayed glycoprotein levels in each 1/10^th^ of the data (shown by the boxplots in Fig 2B). The Spearman correlation was also calculated between the imputed and immunoassayed glycoprotein levels shown in Fig 2A after using the final imputation models to predict the concentration of each glycoprotein in all 626 DILGOM07 participants with serum NMR and matched glycoprotein assay data (point estimates shown in Fig 2B). The difference between this point estimate and the average Spearman correlation in model training (Fig 2B and S2 Table) indicates the amount of overfitting of each model to the training cohort.

The strong correlation structure in the NMR-metabolite measurements meant that the imputation models in S1 Models were not necessarily unique. Re-running the model training procedure led to imputation models comprising different features but with similar accuracy to that shown in Fig 2 and similar hazard ratio estimates as shown in S2 Fig.

### Electronic health record analysis

Electronic health records were obtained and collated for individuals participating in the DILGOM07 and FINRISK97 studies as described in reference [5]. Briefly, electronic health records were obtained from the Finnish National Hospital Discharge Register and the Finnish National Causes-of-Death Register for individuals in DILGOM07 and FINRISK97 from 1987–2015. Electronic health records from 1987–1995 were encoded according to the International Classification of Diseases (ICD) 9^th^ revision (ICD-9) format, and converted to the 10^th^ revision format (ICD10) to match the encoding of records from 1996–2015 using the scheme provided by the Diagnosis Code Set General Equivalence Mappings from the Center for Disease Control in the United States of America (ftp://ftp.cdc.gov/pub/Health_Statistics/NCHS/Publications/ICD10CM/2011/), and were verified using the National Data Policy Group mapping scheme from the New Zealand Ministry of Health (http://www.health.govt.nz/system/files/documents/pages/masterf4.xls). Diagnoses with a mismatch of the first 3 digits in the ICD10 code between the two conversion protocols were verified manually.

Electronic health records were aggregated into distinct disease outcomes for each individual, each comprising an ICD10 disease grouping or ICD10 code at three-digit accuracy. Records were aggregated into incident and prevalent cases for each outcome for each individual. Incident cases comprised the first event (either hospital discharge diagnosis or mortality) in an 8-year follow-up from cohort baseline, chosen to match the maximum follow-up time for DILGOM07. Prevalent cases indicated whether an individual had any event for that outcome from 1987 to baseline (20 years for DILGOM07 and 10 years for FINRISK97), the maximum retrospective period available for the analysis. Main and side diagnoses were treated equally when aggregating electronic health records into incident and prevalent cases of each outcome.

Imputed AAT, imputed AGP, imputed HP, and GlycA were separately tested as biomarkers for incidence of each outcome in 4,540 DILGOM07 participants and 7,321 FINRISK97 participants with all model covariates and excluding pregnant women over the 8-year follow-up, and meta-analysed with an inverse-variance weighted fixed-effects model using the metafor R package [54] version 2.0.0 (Figs 3 and 4 and S2–S4 and S3 Table). Imputation of AAT was successful for 4,496 DILGOM07 participants and 7,246 FINRISK97 participants. Imputation of AGP was successful for 4,474 DILGOM07 participants and 7,151 FINRISK97 participants. Imputation of HP was successful for 4,491 DILGOM07 participants and 7,194 FINRISK97 participants. Any imputed glycoprotein measurements that were outside the range of measurements observed in the glycoprotein assays were excluded (0.64–2.58 mg/L for AAT, 362–1,880 mg/L for AGP, and 0.14–3.95 mg/L for HP), and were not imputed for participants where any of the imputation model inputs were missing. Cox proportional hazards models were fit using age as the time scale and adjusting for sex, smoking status, BMI, systolic blood pressure, alcohol consumption, and prevalent disease, as well as citrate, albumin, and VLDL particle size, which were previously identified as biomarkers for 5-year risk of all-cause mortality alongside GlycA levels in FINRISK97 [10]. Each imputed glycoprotein, GlycA, albumin, citrate, BMI, systolic blood pressure, alcohol consumption and the diameter of VLDL particles were log transformed, and standardised (s.d. = 1) in the statistical analyses while current smoking and sex were coded as categorical covariates. Association analyses were performed for all outcomes with ≥ 20 incident cases in both DILGOM07 and FINRISK97 in the subsets of individuals with successfully imputed concentrations of each glycoprotein. Adjustment for prevalent cases was performed where there were ≥ 10 prevalent cases in the respective subsets of individuals prior to baseline. Hazard Ratios were similar when excluding prevalent cases (S4 Fig). In total, AAT, AGP, HP, and GlycA were tested as biomarkers for 351, 347, 350, and 356 outcomes, respectively (S3 Table).

To control for the many related and unrelated hypothesis tests, P-values were adjusted across all outcomes for each biomarker and cohort separately using the Storey-Tibshirani positive False Discovery Rate method [55] using the qvalue package version 2.4.2 in R version 3.2.3. This method is designed to control for multiple correlated tests such as the nested diagnoses and diagnosis categories tested in this study. We considered any glycoprotein–outcome association to be significant and replicable where its FDR adjusted P-value was < 0.05/3 (Bonferroni correcting the significance threshold of 0.05 for the three glycoproteins) in DILGOM07, FINRISK97, and in the meta-analysis (Figs 3 and S2).

Sensitivity analysis to CRP was performed by fitting Cox proportional hazard models with CRP as an additional covariate (S3 Fig). Hazard ratios were combined in inverse-variance weighted meta-analysis. Sensitivity analysis to prevalent disease adjustment was performed by fitting Cox proportional hazards models in the subset of individuals without any prevalent cases of each outcome using the same model parameters and covariates as described above (S4 Fig).

To assess consistency of hazard ratios calculated from the imputed glycoproteins with those from the immunoassayed glycoproteins (S2 Fig), Cox proportional models were fit for all DILGOM07 participants with immunoassayed glycoproteins (N = 630 for AAT and AGP, N = 626 for HP), and also for the predicted glycoprotein concentrations in the 615 DILGOM07 participants used to train the imputation models. In each case, analyses were restricted to the 46 outcomes with 20 or more events in the respective subsets of DILGOM07.

### Gene expression analysis

To identify pathways associated with AAT levels we used GSEA [30,31] (Java application version 2.2.4) to identify pathways enriched for genes differentially expressed with respect to AAT levels in DILGOM07. We tested enrichment for AAT-associated differential expression in collections of curated gene sets available from the Molecular Signatures Database (MSigDB) (http://software.broadinstitute.org/gsea/msigdb/collections.jsp, accessed May 25^th^ 2017). Specifically, we tested enrichment in the MSigDB Hallmark gene sets [56]; GO biological process, molecular function, and cellular compartment ontologies [57,58]; Kyoto Encyclopedia of Genes and Genomes (KEGG) pathways [59]; and Reactome pathways [60]. Gene sets were tested for enrichment in each collection separately. The Pearson correlation metric was used within GSEA to rank genes by their association with AAT. Age and sex adjusted probe expression levels and age- and sex- adjusted log-transformed AAT levels were provided as input since GSEA does not allow for adjustment of covariates. Expression levels for genes with multiple probes were obtained by taking the highest probe expression in each sample (performed by the GSEA software). After collapsing multiple probes, there were 30,281 genes in total. GSEA calculated an enrichment score for each gene set by taking the maximum of a running-sum statistic [30,31]. This running-sum statistic was calculated by iterating through all genes in descending order by AAT correlation, incrementing the running-sum statistic by a gene’s correlation with AAT if it appears in the gene set of interest, and decrementing the running-sum statistic by 1/30,281 otherwise. Normalised enrichment scores and enrichment score p-values were obtained through a permutation test procedure [30,31] in which samples were shuffled 1,000 times. Normalised enrichment scores were calculated as the enrichment score divided by the average enrichment score for the corresponding gene set across the 1,000 permutations. Permutation test P-values were Benjamini-Hochberg FDR adjusted for multiple testing in each gene set collection separately. We considered any gene set to be significantly enriched for genes either up- or down-regulated with respect to increasing AAT levels where the enrichment FDR adjusted P<0.05 (S5 Table).

To identify functionally related gene sets in whole blood associated with AAT, linear regression models were fit between immunoassayed AAT levels and summary expression profiles of 20 replicable gene coexpression network modules that we previously identified in DILGOM07 [15,32–34] and replicated in an independent cohort [34] (S6 Table) and 346 blood transcriptome modules identified by Li *et al*. using 30,000 blood transcriptomes across 500 studies [35] (S8 Table). Summary expression profiles for each module were calculated as the eigenvector of the first principle component of each module’s expression [61] in DILGOM07. In DILGOM07, 518 participants had matched AAT immunoassay data and transcriptome-wide gene expression profiling. Regression models were adjusted for age and sex. An association between our replicable whole blood modules [15,32–34] and AAT was considered significant where P<0.0025 (Bonferroni adjusted significance threshold for the 20 tested coexpression modules). Associations between Li *et al*.’s blood transcriptome modules and AAT were considered significant where P < 1.44×10^−4^ (Bonferroni adjusted significance threshold for the 346 tested modules).

Identification of our 20 replicable gene coexpression network modules in DILGOM07 was performed using the WGCNA R package [62] and their network topology tested for replication in an independent cohort using the NetRep R package [61], described in full in reference [34]. Here, we also report biological function for 12 of these modules we had not yet characterised for previous publications (S7 Table). Characterisation of each module’s biological function was performed as previously described [34]. First, a core set of genes for each module was defined through a permutation test of module membership. For each module, each probe’s correlation with the module’s summary expression profile was compared to a null distribution of membership scores obtained by calculating the correlation between the module’s summary expression profile and all microarray probes that did not cluster into that module. The membership permutation test p-values were Benjamini-Hochberg FDR adjusted across all probes within the module, and probes with FDR adjusted P<0.05 were considered core module probes robust to the network-discovery clustering parameters. Over-representation analysis of Gene Ontology (GO) biological process terms [57,58] in each module’s gene set was performed using GOrilla [63], and all nominally significant GO terms are reported in S7 Table. REVIGO [64] was used to measure the semantic similarity of these GO terms, ranked by P-value, semantic uniqueness, and dispensability (redundancy).

## Supporting information

S1 Fig Glycoprotein imputation model training and selection in the 615 DILGOM07 participants with complete NMR metabolite measurements and matched glycoprotein data (N = 611 for HP). Each plot shows the lasso model tuning in the 10-fold cross validation model training procedure (Methods). Grey bars show the range and red points show the average of the mean-square error (MSE) across the 10 test folds for each of the 100 lasso regression λ penalties (x axes). Numbers on the top axes correspond to the number of features selected for inclusion given the corresponding λ penalty. Age, sex, BMI, and 149 metabolic measures by NMR (S1 Table) were considered as candidate features for each imputation model. For each glycoprotein, the black dashed line indicates the imputation model with smallest average MSE across the 10 test folds during model training. The coloured dashed line indicates the selected model (detailed in S1 Models); the simplest model within 1 standard error of the model with the smallest average MSE. Note the MSE cannot be compared between the different glycoproteins since their range of concentrations differ.
(TIF)

S2 Fig Forest plots for glycoprotein associated risks of disease and mortality from Fig 3 comparing hazard ratios from meta-analysis (green) to hazard ratios calculated in each of DILGOM07 (blue) and FINRISK97 (purple) (Methods). For comparison, hazard ratios calculated from the immunoassayed glycoprotein concentrations (red) and calculated from the predicted glycoprotein concentrations in the 615 DILGOM07 participants used to train the imputation models (yellow; labelled “Training”) are also shown provided there were >20 incident events in the diagnosis category (Methods). The number of incident events for each outcome in each cohort are shown to the left of each hazard ratio. FDR-adjusted p-values (Methods) are shown to the right of each hazard ratio. Solid hazard ratios and 95% confidence intervals indicate a significant association (FDR < 0.05/3). Significant and replicable associations (FDR < 0.05/3 in DILGOM07, FINRISK97 and meta-analysis) have highlighted backgrounds. The alphanumeric codes in the square brackets indicate the ICD10 codes or disease categories for each diagnosis. Hazard ratios for all outcomes and all cohorts are provided in S3 Table.
(TIF)

S3 Fig Sensitivity analysis of glycoprotein biomarker associations to CRP adjustment for A) outcomes with significant replicable and replicable associations for each biomarker, and B) across all outcomes with ≥ 20 events in both DILGOM07 and FINRISK97. In both A) and B) each plot compares the hazard ratios conferred per standard deviation increase of the glycoprotein (x-axes) to hazard ratios conferred per standard deviation increase of the glycoprotein adjusted for CRP (y-axes) in meta-analysis of DILGOM07 and FINRISK97 (Methods). Data are shown on a square root scale. The grey dashed diagonal line indicates the location where a hazard ratio would fall if it was unchanged after CRP adjustment. Light grey crosses centred on each hazard ratio represent the 95% confidence intervals for the hazard ratio (horizontal bars) and for the hazard ratio adjusted for CRP (vertical bars). In A) hazard ratios are coloured according to the legend. The coloured line in each plot in B) shows the line of best fit (linear regression) of the CRP-adjusted hazard ratios on the hazard ratios without CRP adjustment, indicating the overall attenuation by CRP across all outcomes. In B) 95% confidence intervals with an upper limit ≥ 2.5 or a lower limit ≤ 0.5 are truncated on the plot.
(TIF)

S4 Fig Sensitivity analysis of glycoprotein biomarker associations to prevalent disease. Each plot compares the hazard ratios (diamonds) calculated when adjusting for prevalent case status as a covariate (x-axes) to hazard ratios calculated excluding individuals with any prevalent cases for each outcome (y-axes) in meta-analysis of DILGOM07 and FINRISK97. Light grey crosses centred on each hazard ratio represent the 95% confidence intervals for the hazard ratio calculated when adjusting for prevalent cases as a covariate (horizontal bars) and for the hazard ratio calculated excluding prevalent cases of each outcome (vertical bars). The grey dashed diagonal line indicates the location where hazard ratios should fall if their estimates are identical in the different models. Red diamonds indicate outcomes significantly associated with each biomarker in Fig 3.
(TIF)

S1 Table Serum NMR measurements that were included as inputs to the lasso regression models used to identify the glycoprotein imputation models. Derived ratios were excluded from the analyses (Methods).
(XLSX)

S2 Table Glycoprotein imputation model accuracy assessed in the 626 DILGOM07 participants with matched glycoprotein assay, serum NMR-metabolite measures, age, sex, and BMI. The first row shows the Spearman’s rank correlation coefficient (ρ) calculated between the predicted and observed glycoprotein measurements in Fig 2A. The second row shows its mean ± standard deviation in the 10-fold cross-validation procedure used to train each imputation model (Fig 2B).
(XLSX)

S3 Table Hazard ratios for all tested outcomes. Rows are organised by biomarker, then by ICD10 diagnosis, then by the cohort the association between the biomarker and ICD10 diagnosis was tested in. ICD10: The code for the diagnosis or range of codes for the diagnosis category. Diagnosis: name of the ICD10 diagnosis or diagnosis category. Cohort: association test cohort; “Meta-analysis”: meta-analysis of the hazard ratios calculated in the DILGOM07 and FINRISK97 cohorts; “FINRISK97”: samples in FINRISK97 for which each glycoprotein was successfully imputed (or for GlycA all samples with NMR metabolite measurements); “DILGOM07”: samples in DILGOM07 for which each glycoprotein was successfully imputed (or for GlycA all samples with NMR metabolite measurements); “Immunoassay”: association between the glycoprotein measured by immunoassay in the subset of DILGOM07 with immunoassay measurements; “Training”: association between the imputed glycoprotein in the subset of DILGOM07 used to train each imputation model (i.e. with complete NMR data and immunoassay measurements). Samples: the number of samples in each cohort for that biomarker. Events: the number of people diagnosed with that ICD10 code in the 8-year follow-up period. ICD10 diagnoses with < 20 events in each sub-cohort could not be analysed. Prevalent: the number of people diagnosed with that ICD10 code in the 10-year follow-up period; Cox proportional hazard models were adjusted for prevalent cases where there were > 10 prevalent cases. HR: Cox proportional hazard ratio between the biomarker and the ICD10 diagnosis in the corresponding sub-cohort. Models were fit using age as the time scale and adjusting for sex, smoking status, BMI, blood pressure, alcohol consumption, prevalent cases, and previously identified biomarkers for 5-year risk of all-cause mortality (citrate, albumin, and VLDL particle size). SE: standard error of the hazard ratio. 95% CI: 95% confidence interval. P: P-value. FDR: Storery-Tibshirani adjusted P-value. FDR correction was performed across P-values for each biomarker and cohort separately. Benjamini-Hochberg FDR correction was performed for AGP in the “Training” cohort due to errors arising from the qvalue function for Storery-Tibshirani FDR correction. Sig: FDR corrected P-value is significant at the designated cut-off FDR < 0.05/3 (0.0167). SigRep: Association between the biomarker and ICD10 diagnosis was significant and replicable: FDR < 0.05/3 in the DILGOM07 and FINRISK97 cohorts and in their meta-analysis.
(XLSX)

S4 Table Spearman correlation between GlycA and each glycoprotein. Immunoassayed: Spearman correlation between GlycA and each immunoassayed glycoprotein in the 626 DILGOM07 participants with matched immunoassay and NMR-metabolite measures. Training: Spearman correlation between GlycA and the imputed glycoprotein levels in the 615 DILGOM07 participants used for model training. DILGOM07: Spearman correlation between GlycA and the imputed glycoprotein levels in all 4,540 DILGOM07 participants. FINRISK97: Spearman correlation between GlycA and the imputed glycoprotein levels in all 7,321 FINRISK97 participants.
(XLSX)

S5 Table Curated gene sets significantly enriched for genes associated with AAT. Each sheet corresponds to one of the following collection of pathways or gene sets: Hallmark pathways, KEGG pathways, Reactome pathways, GO biological process terms, GO molecular function terms, and GO cellular compartment terms significantly enriched for AAT-associated differential expression (Methods). Size: number of genes on the Illumina HT-12 array annotated for the corresponding gene set. NES: enrichment score normalised by the average enrichment score for the respective gene set in a permutation procedure with 1,000 permutations (Methods). A positive NES indicates the gene set is enriched for genes upregulated with elevated AAT while a negative NES indicates the gene set is enriched for genes downregulated with elevated AAT. FDR: Benjamini-Hochberg FDR adjusted permutation test P-value for enrichment. FDR correction was performed within each collection separately. Gene sets with FDR<0.05 are shown.
(XLSX)

S6 Table Association between AAT and replicable gene coexpression network modules in DILGOM07. The tested gene coexpression network modules are those that were previously identified in DILGOM07 and found to topologically replicate in an independent cohort study in reference [34]. References are given next to module names referring to the study in which they were first characterised. Modules without references are those first characterised here based on GO term enrichment shown in S7 Table. Linear regression models were fit between log-transformed AAT and each modules summary expression profile (first principal component) adjusting for participant age and sex. Modules were given numeric labels in descending order of module size (40 modules were identified, 20 were found to be topologically replicable). Effect size indicates the change in standard deviations of immunoassayed AAT conferred per standard deviation increase of each module’s coordinated expression adjusting for participant age and sex. 95% CI indicates the 95% confidence interval for the effect size. An association was considered significant (top three modules) where its P-value < 0.0025 (0.05/20, Bonferroni correcting for the total number of modules tested).
(XLSX)

S7 Table Enrichment analysis for reproducible DILGOM07 gene coexpression modules. Gene Ontology (GO) biological process terms significantly over-represented in the core gene set for each gene coexpression module (one sheet per module) along with REVIGO ranking of nominally significant GO terms by information content (Methods). “GO Term”: GO ID for the GO term. “P-value”: P-value from a hypergeometric test. “FDR q-value”: Benjamini-Hochberg FDR adjusted P-value. All GO terms with P < 0.05 are displayed. “N”: number of genes on the Illumina HT-12 array with any GO annotation. “B”: number of genes in the GO term. “n”: number of genes in the module. “b”: number of genes in the module annotated for the corresponding GO term. “frequency”: percentage of array annotated for the corresponding GO term. “uniqueness”: REVIGO measure of semantic uniqueness when compared to all other GO terms. “dispensability”: REVIGO measure of semantic redundancy when compared to its most similar GO term. “eliminated”: 1 or 0 depending on whether the GO Term was considered redundant by the REVIGO analysis. Rows in each sheet are organised by the “eliminated” column, then by P-value.
(XLSX)

S8 Table Association between AAT and 346 blood transcript modules in DILGOM07. The tested network modules are those identified by Li et al. in reference [35]. “size”: number of genes in the module. “in array”: number of genes that could be mapped to the Illumina HT12 array used in DILGOM07. Linear regression models were fit between log-transformed AAT and each modules summary expression profile (first principal component) adjusting for participant age and sex in the 518 DILGOM07 participants with matched gene expression and AAT immunoassay data. “beta”: change in standard deviations of immunoassayed AAT conferred per standard deviation increase of each module’s coordinated expression adjusting for participant age and sex. L95 and U95: lower and upper bounds of the 95% confidence interval.
(XLSX)

S1 Models Summary of imputation models for AAT, AGP, HP, and TF. A) Here, coefficients are rounded to two significant figures. Full precision models are made available through the imputegp R package which can be downloaded and installed from https://github.com/sritchie73/imputegp. Each model predicts the concentration of each glycoprotein on a natural logarithm scale. Concentrations in mg/L are obtained by exponentiating the result. Variables in the imputation models are also required on a natural logarithm scale, with the exceptions of participant Age and Sex. The coding for the Sex variable was 1 for men and 2 for women. The NMR-metabolite measures are described in S1 Table. B) Model coefficients are given after log transformation of each variable and standardisation to their mean and standard deviation in DILGOM07, i.e. coefficients indicate relative contribution to determining the concentration of the glycoprotein. Variables are listed from left to right in descending order of their relative contribution in both A) and B).
(DOCX)
