# Supplementary figures and images for: Elevated serum alpha-1 antitrypsin is a major component of GlycA-associated risk for future morbidity and mortality
Source: PLoS One. 2019 Oct 23;14(10):e0223692. doi: 10.1371/journal.pone.0223692 (PMC6808431; doi:10.1371/journal.pone.0223692)

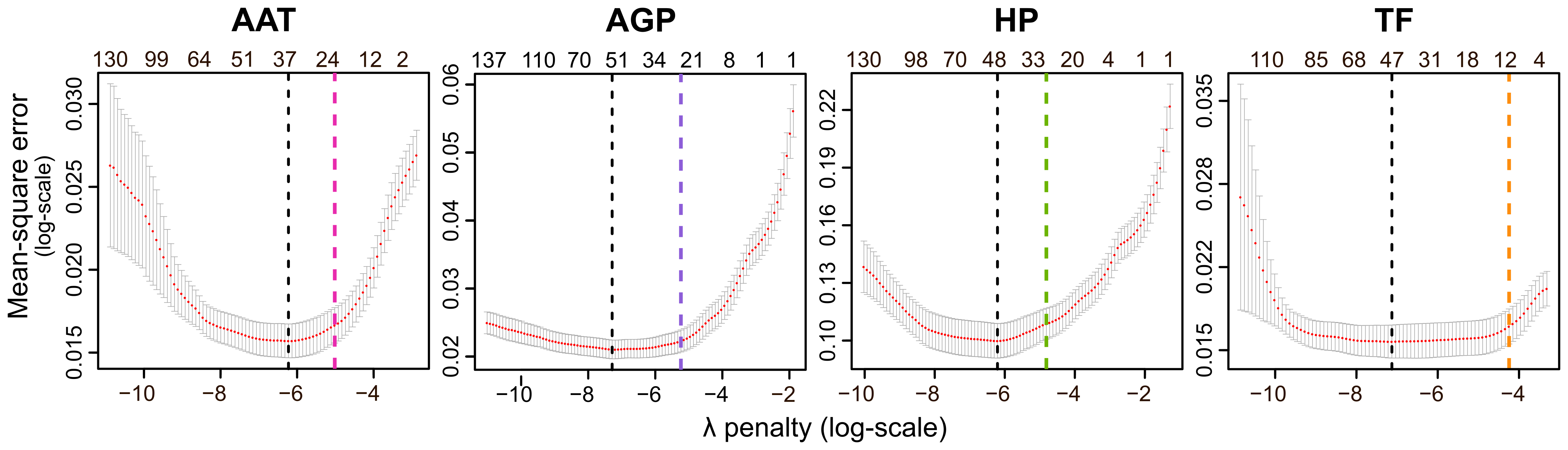

Supplement: S1 Fig — Each plot shows the lasso model tuning in the 10-fold cross validation model training procedure (Methods). Grey bars show the range and red points show the average of the mean-square error (MSE) across the 10 test folds for each of the 100 lasso regression λ penalties (x axes). Numbers on the top axes correspond to the number of features selected for inclusion given the corresponding λ penalty. Age, sex, BMI, and 149 metabolic measures by NMR (S1 Table) were considered as candidate features for each imputation model. For each glycoprotein, the black dashed line indicates the imputation model with smallest average MSE across the 10 test folds during model training. The coloured dashed line indicates the selected model (detailed in S1 Models); the simplest model within 1 standard error of the model with the smallest average MSE. Note the MSE cannot be compared between the different glycoproteins since their range of concentrations differ. (TIF) [file pone.0223692.s001.tif]

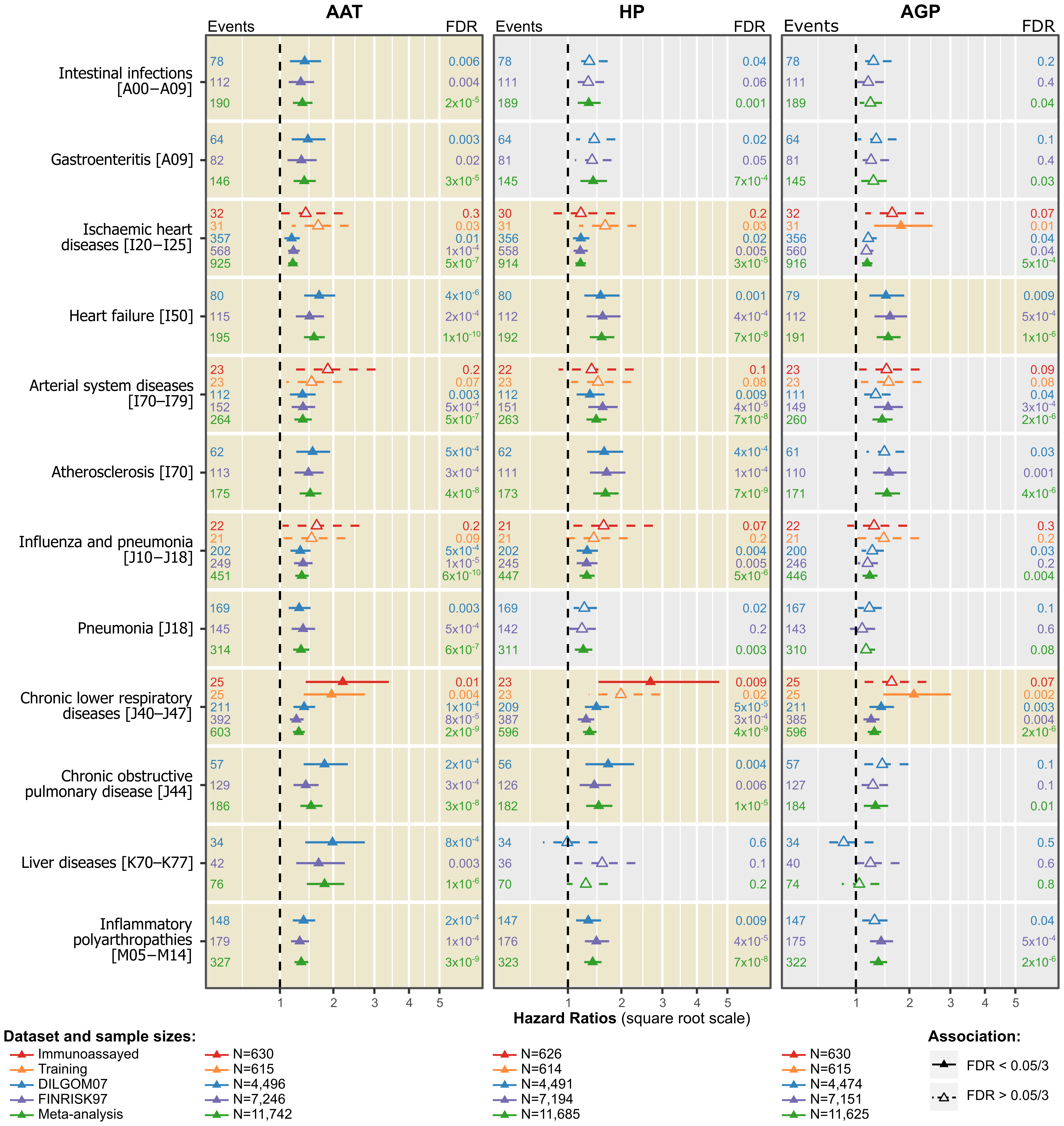

Supplement: S2 Fig — For comparison, hazard ratios calculated from the immunoassayed glycoprotein concentrations (red) and calculated from the predicted glycoprotein concentrations in the 615 DILGOM07 participants used to train the imputation models (yellow; labelled “Training”) are also shown provided there were >20 incident events in the diagnosis category (Methods). The number of incident events for each outcome in each cohort are shown to the left of each hazard ratio. FDR-adjusted p-values (Methods) are shown to the right of each hazard ratio. Solid hazard ratios and 95% confidence intervals indicate a significant association (FDR < 0.05/3). Significant and replicable associations (FDR < 0.05/3 in DILGOM07, FINRISK97 and meta-analysis) have highlighted backgrounds. The alphanumeric codes in the square brackets indicate the ICD10 codes or disease categories for each diagnosis. Hazard ratios for all outcomes and all cohorts are provided in S3 Table. (TIF) [file pone.0223692.s002.tif]

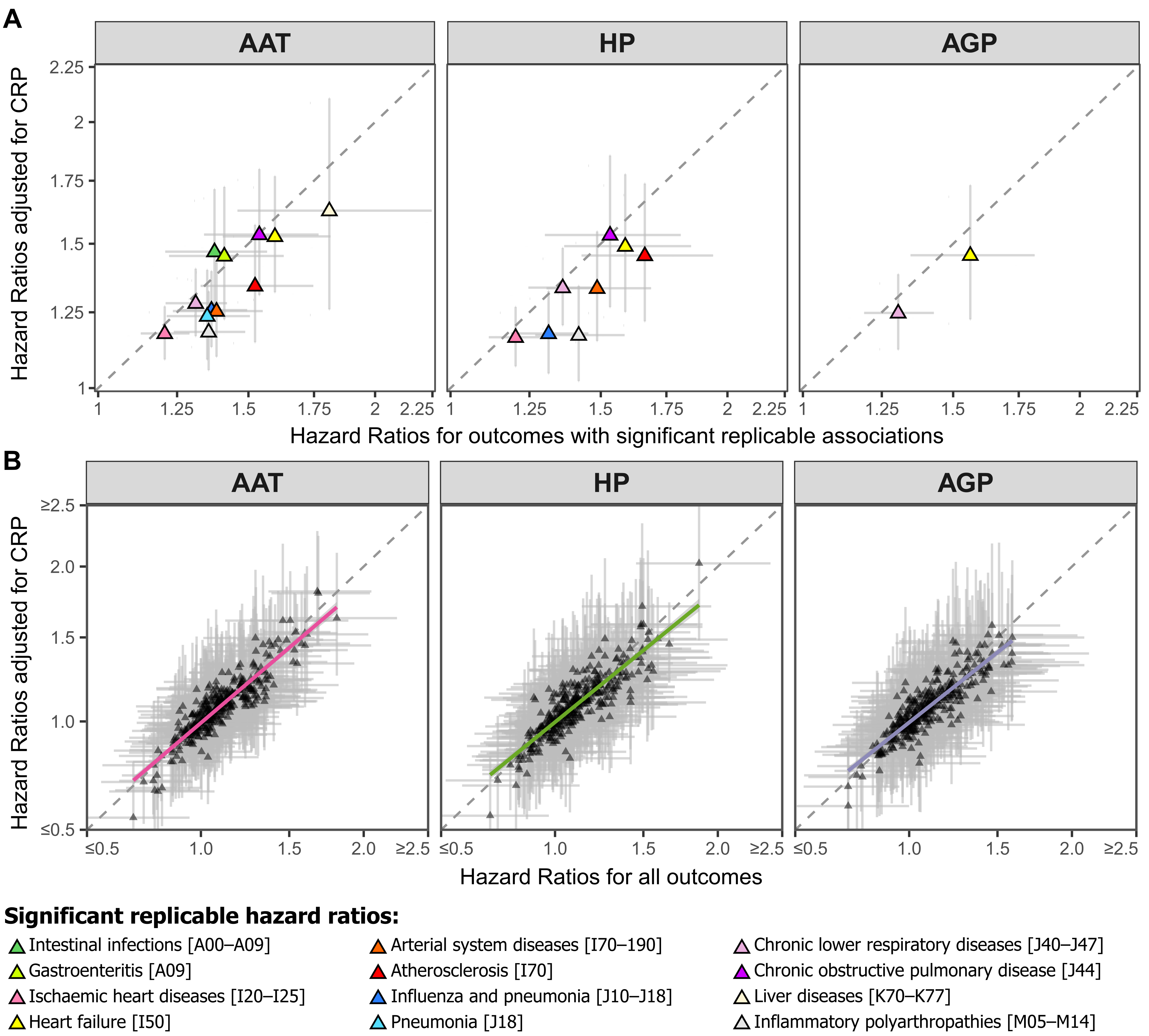

Supplement: S3 Fig — Sensitivity analysis of glycoprotein biomarker associations to CRP adjustment for A) outcomes with significant replicable and replicable associations for each biomarker, and B) across all outcomes with ≥ 20 events in both DILGOM07 and FINRISK97. In both A) and B) each plot compares the hazard ratios conferred per standard deviation increase of the glycoprotein (x-axes) to hazard ratios conferred per standard deviation increase of the glycoprotein adjusted for CRP (y-axes) in meta-analysis of DILGOM07 and FINRISK97 (Methods). Data are shown on a square root scale. The grey dashed diagonal line indicates the location where a hazard ratio would fall if it was unchanged after CRP adjustment. Light grey crosses centred on each hazard ratio represent the 95% confidence intervals for the hazard ratio (horizontal bars) and for the hazard ratio adjusted for CRP (vertical bars). In A) hazard ratios are coloured according to the legend. The coloured line in each plot in B) shows the line of best fit (linear regression) of the CRP-adjusted hazard ratios on the hazard ratios without CRP adjustment, indicating the overall attenuation by CRP across all outcomes. In B) 95% confidence intervals with an upper limit ≥ 2.5 or a lower limit ≤ 0.5 are truncated on the plot. (TIF) [file pone.0223692.s003.tif]

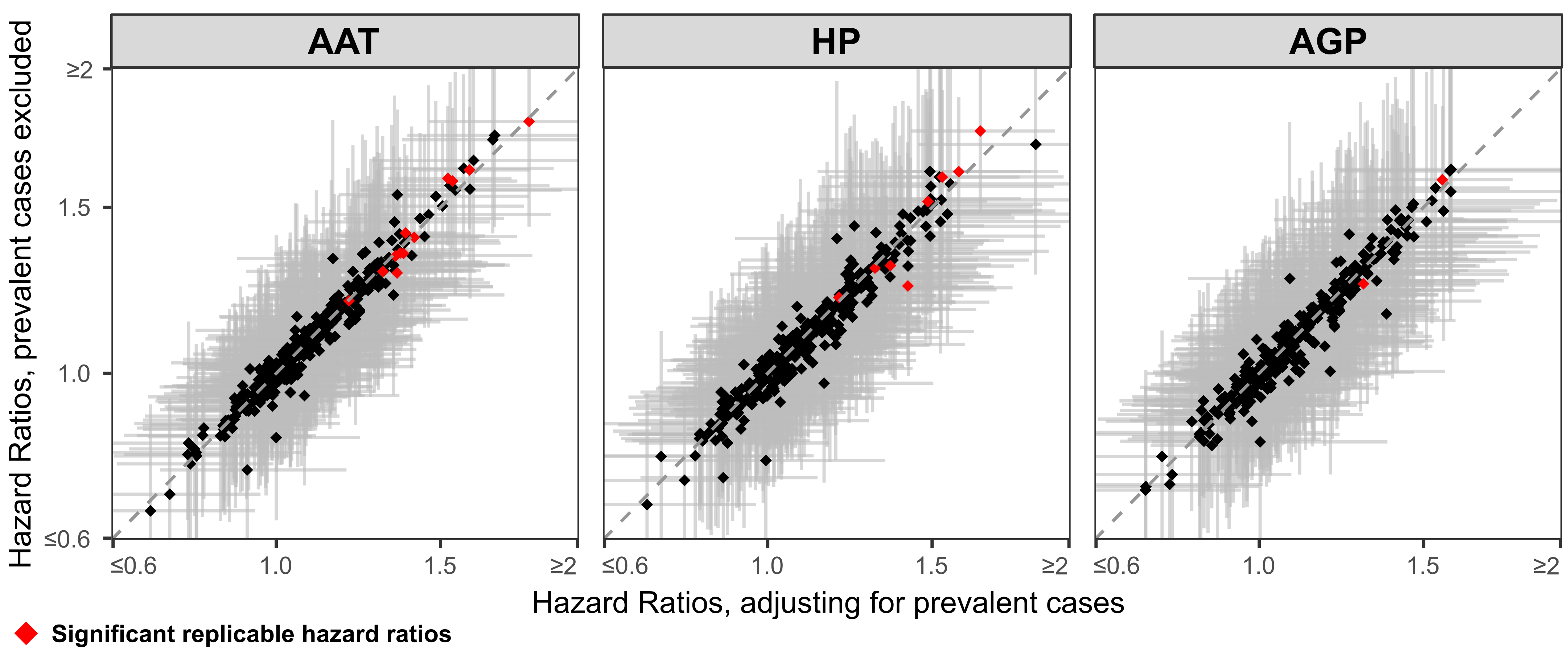

Supplement: S4 Fig — Each plot compares the hazard ratios (diamonds) calculated when adjusting for prevalent case status as a covariate (x-axes) to hazard ratios calculated excluding individuals with any prevalent cases for each outcome (y-axes) in meta-analysis of DILGOM07 and FINRISK97. Light grey crosses centred on each hazard ratio represent the 95% confidence intervals for the hazard ratio calculated when adjusting for prevalent cases as a covariate (horizontal bars) and for the hazard ratio calculated excluding prevalent cases of each outcome (vertical bars). The grey dashed diagonal line indicates the location where hazard ratios should fall if their estimates are identical in the different models. Red diamonds indicate outcomes significantly associated with each biomarker in Fig 3. (TIF) [file pone.0223692.s004.tif]
