# Supplementary material for: Elevated serum alpha-1 antitrypsin is a major component of GlycA-associated risk for future morbidity and mortality
Source: PLoS One. 2019 Oct 23;14(10):e0223692. doi: 10.1371/journal.pone.0223692 (PMC6808431; doi:10.1371/journal.pone.0223692)
Supplement: S1 Models — A) Here, coefficients are rounded to two significant figures. Full precision models are made available through the imputegp R package which can be downloaded and installed from https://github.com/sritchie73/imputegp. Each model predicts the concentration of each glycoprotein on a natural logarithm scale. Concentrations in mg/L are obtained by exponentiating the result. Variables in the imputation models are also required on a natural logarithm scale, with the exceptions of participant Age and Sex. The coding for the Sex variable was 1 for men and 2 for women. The NMR-metabolite measures are described in S1 Table. B) Model coefficients are given after log transformation of each variable and standardisation to their mean and standard deviation in DILGOM07, i.e. coefficients indicate relative contribution to determining the concentration of the glycoprotein. Variables are listed from left to right in descending order of their relative contribution in both A) and B). (DOCX) [file pone.0223692.s013.docx]

**A)**

$$\text{AAT}= -11+0.68 \text{Gly}\text{cA}-0.094 \text{FAw3}-0.8 \text{VLDL-D}+0.37 \text{HDL3-C}+4.2 \text{LDL-D}+0.14 \text{Phe}-0.08 \text{Leu}-0.064 \text{ApoB}-0.19 \text{Alb}-0.045 \text{Tyr}+0.019 \text{bOHBut}-0.045 \text{BMI}-0.043 \text{Ala}+0.011 \text{L-HDL-TG }-0.022 \text{Ile}-0.025 \text{Ace}-0.038 \text{His}+0.0024 \text{HDL-TG}$$

$$\text{AGP}=5.7+1.7 \text{GlycA}-0.34 \text{TotFA}+0.22 \text{IDL-FC}+0.043 \text{L-HDL-FC}-0.22 \text{His}-0.092 \text{HDL-TG}+0.11 \text{BMI}-0.16 \text{S-HDL-FC}-0.057 \text{S-LDL-TG}-0.023 \text{bOHBut}-0.062 \text{LA}+0.066 \text{S-HDL-CE}-0.055 \text{Lac}-0.021 \text{S-VLDL-TG}-0.042 \text{Ace}-0.052 \text{Cit}-0.038 \text{SFA}-0.049 \text{Ala}+0.0013 \text{XXL-VLDL-CE}+0.014 \text{Glol}+0.00017 \text{Age}+0.0084 \text{Crea}+0.0061 \text{Gly}$$

$$\text{HP}=0.53+3.0 \text{GlycA}-0.82 \text{LA}+0.36 \text{IDL-FC}+0.48 \text{SM}-0.26 \text{FAw3}-0.29 \text{HDL-TG}-0.0068 \text{S-VLDL-CE}+0.003 \text{Age}-0.75 \text{Alb}-0.13 \text{Ile}-0.25 \text{Cit}-0.89 \text{VLDL-D}-0.13 \text{Leu}+0.13 \text{Val}+0.0066 \text{L-VLDL-CE}+0.084 \text{Pyr}-0.084 \text{Lac}-0.094 \text{Gln}+0.042 \text{M-HDL-FC}-0.011 \text{XL-HDL-TG}+0.0089 \text{XL-HDL-PL}-0.048 \text{His}-0.026 \text{Tyr}-0.024 \text{BMI}-0.0037 \text{L-HDL-TG}-0.004 \text{PUFA}+0.00056 \text{S-LDL-FC}$$

$$\text{TF}=1.1+0.14 \text{GlycA}+0.032 \text{Sex}-0.0010 \text{Age}+0.090 \text{S-HDL-FC}-0.037 \text{Ace}+0.039 \text{Ala}+0.024 \text{SFA}+0.013 \text{His}-0.0097 \text{Gln}$$

**B)**

$$\text{AAT}= 0.54 \text{GlycA}-0.17 \text{FAw3}-0.16 \text{VLDL-D}+0.14 \text{HDL3-C}+0.11 \text{LDL-D}+0.11 \text{Phe}-0.096 \text{Leu}-0.081 \text{ApoB}-0.058 \text{Alb}-0.057 \text{Tyr}+0.052 \text{bOHBut}-0.045 \text{BMI}-0.036 \text{Ala}+0.036 \text{L-HDL-TG}-0.035 \text{Ile}-0.033 \text{Ace}-0.027 \text{His}+0.0034 \text{HDL-TG}$$

$$\text{AGP}=0.93 \text{GlycA}-0.27 \text{TotFA}+0.26 \text{IDL-FC}+0.12 \text{L-HDL-FC}-0.11 \text{His}-0.091 \text{HDL-TG}+0.08 \text{BMI}-0.079 \text{S-HDL-FC}-0.073 \text{S-LDL-TG}-0.05 \text{bOHBut}-0.048 \text{LA}+0.045 \text{S-HDL-CE}-0.043 \text{Lac}-0.043 \text{S-VLDL-TG}-0.04 \text{Ace}-0.037 \text{Cit}-0.035 \text{SFA}-0.032 \text{Ala}+0.026 \text{XXL-VLDL-CE}+0.022 \text{Glol}+0.013 \text{Age}+0.013 \text{Crea}+0.0041 \text{Gly}$$

$$\text{HP}=0.84 \text{GlycA}-0.29 \text{LA}+0.21 \text{IDL-FC}+0.18 \text{SM}-0.16 \text{FAw3}-0.14 \text{HDL-TG}-0.088 \text{S-VLDL-CE}+0.087 \text{Age}-0.078 \text{Alb}-0.076 \text{Ile}-0.074 \text{Cit}-0.062 \text{VLDL-D}-0.055 \text{Leu}+0.051 \text{Val}+0.048 \text{L-VLDL-CE}+0.04 \text{Pyr}-0.032 \text{Lav}-0.023 \text{Gln}+0.022 \text{M-HDL-FC}-0.014 \text{XL-HDL-TG}+0.012 \text{XL-HDL-PL}-0.012 \text{His}-0.011 \text{Tyr}-0.0086 \text{BMI}-0.004 \text{L-HDL-TG}-0.0014 \text{PUFA}+0.00025 \text{S-LDL-FC}$$

$$\text{TF}=0.13 \text{GlycA}+0.11 \text{Sex}-0.097 \text{Age}+0.08 \text{S-HDL-FC}-0.057 \text{Ace}+0.037 \text{Ala}+0.031 \text{SFA}+0.011 \text{His}-0.0079 \text{Gln}$$
